# Serial Radiographic Evolution and All-Cause Mortality in Older Adults with Isolated Type II Odontoid Fractures: A 15-Year Single-Center Cohort Study

**DOI:** 10.1177/21925682261507648

**Published:** 2026-11-06

**Authors:** Jan Gewiess, Domas Bubulis, Samuel Friedrich Schaible, Moritz Caspar Deml, Thomas Lustenberger, Christian Tinner

**Affiliations:** 1 Department of Orthopaedic Surgery and Traumatology, Inselspital, 27252 Bern University Hospital, University of Bern, Bern, Switzerland

**Keywords:** odontoid fracture, type II odontoid fracture, geriatric spine trauma, cervical spine, mortality, non-operative management, cervical alignment, spinal trauma

## Abstract

**Study Design:**

Retrospective cohort study.

**Objectives:**

To characterize serial radiographic evolution after operative and non-operative management of isolated type II odontoid fractures in older adults and examine all-cause mortality.

**Methods:**

Adults aged ≥65 years with isolated type II odontoid fractures treated at a Level I trauma center between 2010 and 2025 were included. Serial fracture displacement, angulation, and cervical alignment were assessed through 6 months. Mixed-effects models evaluated longitudinal changes, with fixation-technique comparisons explored within the operative subgroup. All-cause mortality from definitive treatment initiation was assessed using Kaplan–Meier analysis and age-adjusted Cox regression.

**Results:**

Eighty-six patients were included: 33 operative and 53 non-operative. Among patients with available follow-up imaging, operative reduction was maintained, and no statistically significant progressive displacement or angulation was detected after non-operative management. No significant timepoint-by-technique interaction was detected for displacement (p=0.092) or angulation (p=0.823). Fifty deaths occurred. Non-operative management was not significantly associated with mortality compared with operative treatment (HR 0.93, 95% CI 0.51–1.70; p=0.806), whereas increasing age was independently associated with mortality (HR 1.09 per year, 95% CI 1.05–1.13; p<0.0001). Including calendar year did not materially alter the treatment estimate (HR 0.88, 95% CI 0.48–1.63; p=0.688).

**Conclusions:**

Through 6 months, operative reduction was maintained and no statistically significant radiographic progression was detected after non-operative management among patients with available imaging. All-cause mortality was associated with age, whereas no significant association with treatment strategy was detected. These findings do not establish treatment superiority or equivalence.

## Introduction

Odontoid Type II fractures are the predominant odontoid fracture subtype in older adults.^1^ The optimal treatment strategy remains controversial.^2^ Operative treatment may provide fracture reduction, mechanical stabilization, and higher fusion rates, whereas non-operative treatment may avoid perioperative morbidity and can result in stable fibrous nonunion.^3^ Reported mortality outcomes after operative and non-operative treatment remain conflicting and are difficult to interpret owing to selection bias as surgical patients are often younger and less frail.^2,4,5^

Although numerous studies have compared mortality and fusion after operative and non-operative treatment, considerably less research has focused on serial radiographic evolution following treatment.^3,6^ In particular, it remains unclear whether operative reduction is maintained during follow-up and whether non-operative treatment is associated with progressive displacement, angulation, or cervical alignment deterioration over time. This information is clinically relevant because treatment decisions in older adults often balance mechanical stability, operative risk, and patient-specific goals rather than radiographic union alone.

The primary aim of this study was to characterize serial radiographic evolution following operative and non-operative treatment of isolated type II odontoid fractures in adults aged ≥65 years. We specifically assessed whether operative reduction was maintained during follow-up and whether non-operative management was associated with progressive fracture displacement, angulation, or deterioration in cervical alignment. Secondary aims were to compare age and comorbidity scores between definitive treatment groups and to examine whether definitive treatment strategy was associated with all-cause mortality after adjustment for age.

## Methods

### Study Design and Setting

This retrospective single-center cohort study was approved by the local ethics committee and conducted in accordance with the Declaration of Helsinki. The study was performed at a single Level I trauma center using an institutional imaging database search and is reported in accordance with the Strengthening the Reporting of Observational Studies in Epidemiology (STROBE) statement. Given the nonrandomized design, all comparative analyses were interpreted as observational. No formal sample-size calculation was performed; all eligible patients during the study period were included.

### Participants

Older adults presenting to our institution with an acute Anderson and D’Alonzo type II odontoid fracture between 1 January 2010 and 1 January 2025 were screened for eligibility. Inclusion criteria were age ≥65 years and an isolated odontoid type II fracture according to the Anderson and D’Alonzo classification,^7^ and neurologically intact status at presentation. Patients were excluded if they had other C2 fracture patterns, including odontoid type I or III fractures, hangman fractures, or vertebral body fractures, as well as additional cervical spine injuries such as occipital condyle or atlas fractures, pathological fractures, prior odontoid fractures, missing CT, or insufficient data. Baseline comorbidity burden was assessed using the Charlson Comorbidity Index (CCI) and the van Walraven algorithm for the Elixhauser Comorbidity Index (ECI).^8,9^

### Treatment Strategy

Treatment allocation was not randomized, and no prespecified radiographic threshold mandated either strategy. Decisions reflected the clinical and radiographic assessment of the treating spine surgeon. Operative management was generally selected for unacceptable fracture displacement or angulation, persistent severe pain, or failure of initial collar treatment, provided that the patient’s physiological condition and treatment goals supported surgery. Non-operative management was generally selected when alignment was considered acceptable or when operative risk, frailty, limited physiological reserve, or patient preference favored collar treatment.

Patients who were initially treated non-operatively but subsequently underwent surgery were analyzed according to definitive treatment received. This as-treated approach was chosen because the primary objective was to characterize outcomes after the final management pathway rather than to estimate the causal effect of initial treatment assignment. The frequency of crossover was reported descriptively. Radiographs obtained at approximately 1 week, 6 weeks, and 6 months after definitive treatment initiation were included in the longitudinal analyses.

Operative management consisted of anterior cannulated odontoid screw fixation or posterior C1–2 fixation using the Harms technique. Anterior fixation was performed by placing cannulated screws across the fracture into the odontoid fragment. The number of screws varied according to fracture morphology and surgeon preference. Posterior fixation used C1 lateral-mass and C2 pedicle or pars screws connected by rods. Bone grafting was performed selectively.

Non-operative management followed a standardized institutional protocol consisting of semi-rigid cervical collar immobilization for 24 hours per day for 6 weeks, after which collar use was discontinued. CT-based assessment of osseous union and dynamic flexion–extension radiographs were not routinely performed at our institution and were therefore unavailable for systematic analysis.

### Radiographic Measurements

Fracture displacement was measured as the distance between a tangent drawn along the anterior aspect of the odontoid fragment and the anterior cortex of the C2 vertebral body. Fracture angulation was measured as the angle between a tangent drawn along the posterior aspect of the odontoid fragment and the posterior cortex of the C2 body.^10^ Odontoid incidence was defined as the angle between a line perpendicular to the C2 endplate at its midpoint and a line connecting this point to the center of the odontoid process. Odontoid tilt was measured as the angle between the line connecting the midpoint of the C2 endplate to the center of the odontoid process and the vertical axis. C2 slope was defined as the angle between the C2 endplate and a horizontal reference line.^11^

Displacement and angulation were retained as signed measurements. The sign describes measurement direction rather than absolute magnitude. Absolute displacement values were additionally summarized to characterize injury severity descriptively. Injury radiographs were distinguished from the approximately 1-week, 6-week and 6-month post-treatment assessments. Operative measurements at these follow-up timepoints represent residual alignment after reduction and fixation.

Global and regional alignment parameters were also recorded. The gravity line-to-C2 distance was measured as the horizontal distance between a plumb line dropped from the center of the acoustic meatus and the center of C2 ^11^. Cervical sagittal vertical axis (cSVA) was defined as the distance between a plumb line from the centroid of C2 and the posterosuperior corner of C7. The C0–2 angle was measured between the McGregor line and the inferior endplate of C2, whereas the C2–7 angle was measured between the inferior endplates of C2 and C7. T1 slope was measured as the angle between a horizontal line and the superior endplate of T1. The radiographic measurement techniques are illustrated in Figure 1.

**Figure 1.**
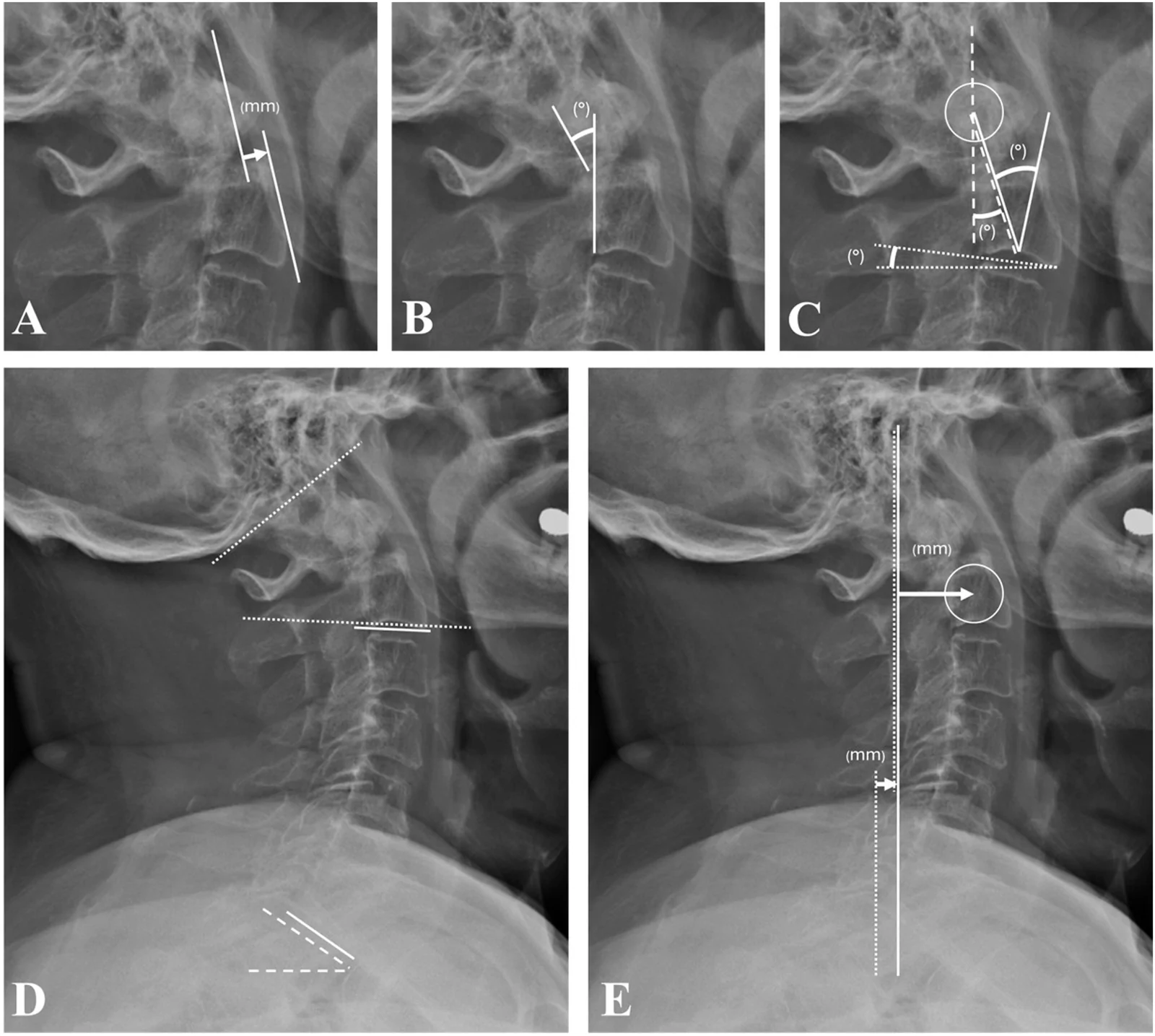
Radiographic measurement techniques on lateral cervical spine radiographs. (A) Fracture displacement. (B) Fracture angulation. (C) Odontoid incidence, odontoid tilt, and C2 slope. (D) C0–2 angle, C2–7 angle, and T1 slope. (E) Gravity line-to-C2 distance and cervical sagittal vertical axis. Linear measurements are expressed in millimeters and angular measurements in degrees. cSVA, cervical sagittal vertical axis

Baseline injury CT parameters were additionally analyzed according to the radiographic framework described by Ogden et al.^12^ The CT distances comprised right and left transverse-ligament attachment-to-odontoid distances [TL-OD (R) and TL-OD (L)]; anterior and posterior atlas-to-odontoid distances [C1 (A)-OD and C1 (P)-OD]; atlas- and axis-to-fracture-point distances [C1 (P)-OFP and C2 (P)-OFP]; the posterior atlas-to-C2 inferior-endplate distance [C1 (P)-OIP]; the anterior atlas-to-C2 lamina distance [C1 (A)-C2P]; and anteroposterior odontoid translation (OD FLIP). The angular parameters were AP-FRAP, defined by the clival apical-ligament attachment, anterior fracture endpoint and posterior C2 body border; Sliding Angle, between the anterior odontoid-fragment and anterior C2 body lines; and the clivus–odontoid angle (CL-OD), measured between lines along the anterior border of the odontoid process and the dorsal border of the clivus. Table 2 provides the anatomical descriptions and units. These baseline CT parameters were distinct from serial lateral-radiographic fracture displacement and angulation.

### Mortality and Survival Analysis

All-cause mortality from definitive treatment initiation to administrative censoring was evaluated as a secondary clinical outcome. Deaths occurring before the administrative censoring date were treated as events. Mortality status was obtained from the institutional electronic medical record, which is linked to each patient’s registered social security number and updated periodically. Cause-of-death information was not systematically available in the records accessible to the study team and was therefore not analyzed.

### Statistical Analysis

All analyses were performed in R version 4.5.1 (R Foundation for Statistical Computing). Continuous variables are presented as mean ± standard deviation or median with interquartile range, and categorical variables as number and percentage. Between-group comparisons were performed using parametric or nonparametric tests as appropriate.

Primary longitudinal analyses of fracture displacement and angulation included the post-treatment assessments at approximately 1 week, 6 weeks, and 6 months. Exploratory analyses of broader cervical alignment additionally incorporated available radiographs. Longitudinal changes were evaluated using mixed-effects models with timepoint as a fixed effect and patient ID as a random intercept. No values were imputed. Mixed-effects models used all available observations, and complete-case analyses were performed as sensitivity analyses. Radiographic availability by treatment group and timepoint is reported in Supplementary Table S3. Holm adjustment was applied within each outcome to the targeted pairwise comparisons to control the family-wise error rate. Complete-case Friedman tests with paired Wilcoxon comparisons were used as sensitivity analyses to assess whether the longitudinal findings were consistent among patients with measurements available at all analyzed timepoints. Within the operative subgroup, exploratory models additionally included fixation technique and its interaction with timepoint. Corresponding analyses using absolute displacement and angulation were performed as sensitivity analyses. Because associations between baseline CT parameters and mortality represented a broader exploratory screening analysis, Benjamini–Hochberg correction was applied across CT parameters within each time horizon and subgroup to control the false discovery rate.

Time to death was calculated from definitive treatment initiation (surgery for operatively managed patients and the beginning of non-operative treatment for non-operatively managed patients) to death or administrative censoring on 1 February 2026. For the time-to-event analyses, the date of definitive treatment initiation was operationally defined as 7 days before the recorded 1-week follow-up radiograph. When that radiograph date was unavailable (n=10; 4 operative and 6 non-operative), the baseline CT date was used as a proxy. Survival was assessed using Kaplan–Meier analysis, the log-rank test, and Cox regression adjusted for age at treatment initiation. The age-adjusted Cox model was repeated in a strict complete-case sensitivity analysis excluding these 10 patients. Calendar year of definitive treatment was added in a sensitivity model. Proportional hazards were assessed using Schoenfeld residuals.

Additional post hoc descriptive analyses summarized cumulative all-cause mortality at 6 and 12 months from the same operationally defined treatment-initiation date (0.5 and 1 year, respectively). Mortality was estimated as 1 minus Kaplan–Meier survival, with pointwise 95% confidence intervals obtained by reversing the log–log survival confidence limits. Cumulative death counts and numbers at risk at each timepoint were also reported. These summaries were not used to infer causal treatment effects.

## Results

### Study Population

A total of 86 patients met the inclusion criteria, including 33 patients managed operatively and 53 managed non-operatively according to definitive treatment received (Figure 2 and Table 1). Median age at definitive treatment initiation was 82.3 years (IQR 74.3–87.3). Four patients in the operative group were initially assigned to non-operative treatment and subsequently crossed over to surgery. These patients were analyzed according to definitive treatment received. Operative treatment consisted of posterior C1–2 fixation using the Harms technique (n=12) or anterior cannulated odontoid screw fixation (n=21). CCI and ECI scores did not differ significantly between groups. Median CCI was 6 (IQR 5–7) in the operative group and 6 (IQR 5–8) in the non-operative group (p=0.164). Median ECI was 11 (IQR 6–14) and 11 (IQR 4–21), respectively (p=0.513). Sex distribution also did not differ significantly between groups (p=0.38).

**Figure 2.**
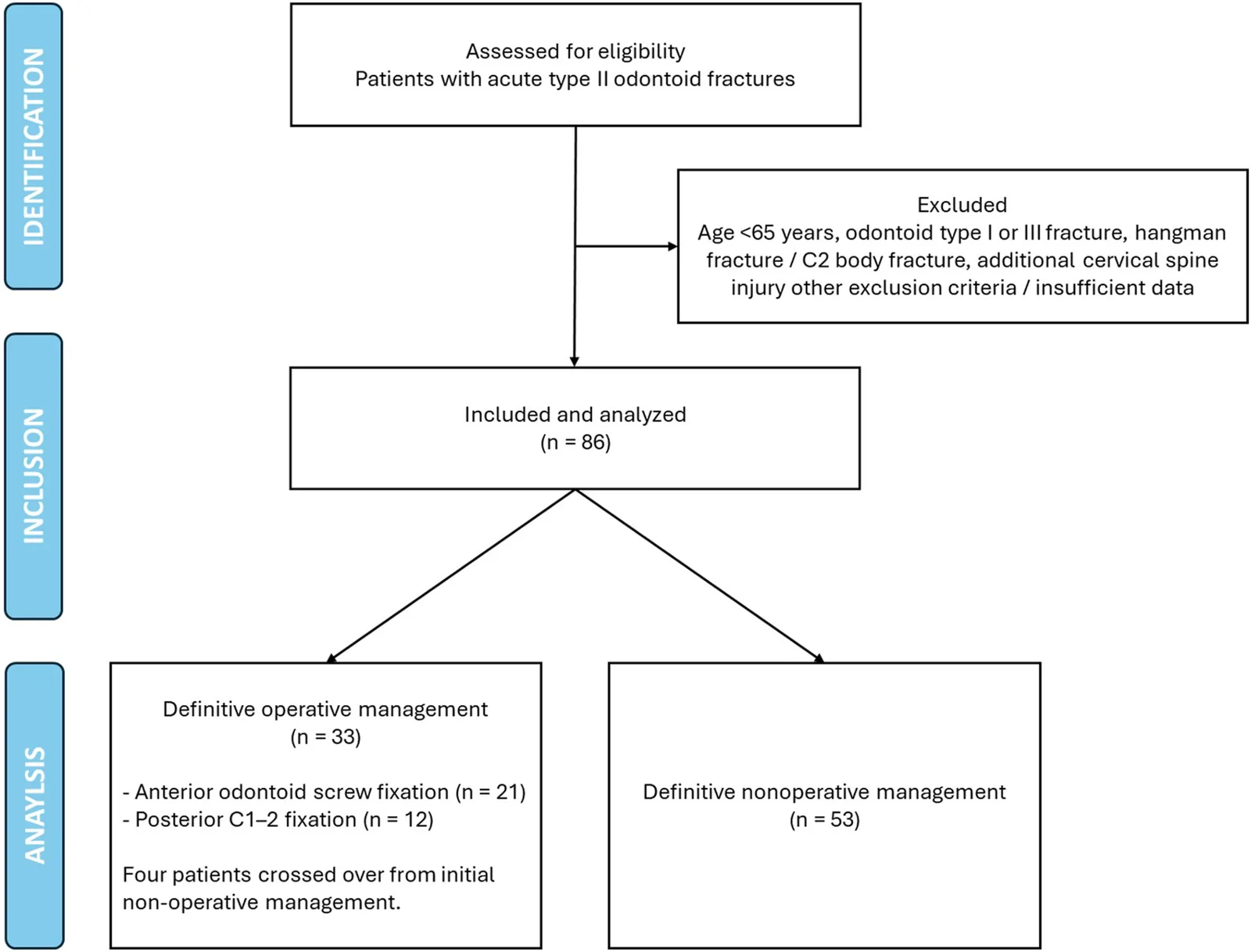
Study flow diagram. Patients were classified according to definitive treatment received. Four patients crossed over from initial non-operative management to surgery and were included in the operative group

**Table 1.** Cohort Characteristics by Definitive Treatment Strategy.

Variable	Operative	Non-operative	p value
Patients, n	33	53	—
Age at definitive treatment initiation, years, median (IQR)	75 (71.6–85.3)	82.6 (78.6–89.2)	0.007
Charlson Comorbidity Index, median (IQR)	6 (5–7)	6 (5–8)	0.164
Elixhauser Comorbidity Index, median (IQR)	11 (6–14)	11 (4–21)	0.513
Observed follow-up duration, months, median (IQR)	49.4 (33.2–90.5)	27.5 (20.2–56.7)	0.013

CCI, Charlson Comorbidity Index; ECI, Elixhauser Comorbidity Index; IQR, interquartile range. Four patients crossed over from initial non-operative management to surgery and were analyzed according to definitive treatment received.

Patients treated operatively were younger than those managed non-operatively, with a median age of 75.0 years (IQR 71.6–85.3) versus 82.6 years (IQR 78.6–89.2), respectively (p=0.007). This age difference was accounted for in the adjusted mortality analysis.

Baseline injury CT findings were broadly similar between definitive treatment groups. The only parameter that differed significantly was anterior-posterior translation of the odontoid process, which was greater in the operative group. All other CT-based radiographic measurements according to Ogden et al^12^ were comparable between groups (Table 2).

**Table 2.** Baseline Injury CT Morphologic Measurements

Variable	Operative	Non-operative	p value
TL-OD (R), mm	9.61 ± 1.44	10.12 ± 1.59	0.126
TL-OD (L), mm	8.95 (8.47–9.93)	9.80 (8.70–11.20)	0.054
C1 (A)-OD, mm	1.30 (1.00–1.80)	1.20 (0.90–1.50)	0.096
C1 (P)-OD, mm	22.05 ± 2.66	21.96 ± 2.63	0.874
C1 (P)-OFP, mm	25.01 ± 3.53	24.69 ± 3.42	0.686
C2 (P)-OFP, mm	21.30 (17.48–23.23)	20.60 (18.40–22.90)	0.845
C1 (P)-OIP, mm	31.27 ± 4.18	29.89 ± 3.51	0.127
C1 (A)-C2P, mm	35.34 ± 4.57	36.10 ± 3.23	0.415
OD FLIP, mm	3.10 (2.35–4.78)	2.00 (1.50–2.80)	0.001*
AP-FRAP, °	26.84 ± 5.69	27.75 ± 3.86	0.429
Sliding Angle, °	10.50 (4.88–20.18)	10.00 (4.60–17.00)	0.982
CL-OD, °	39.77 ± 10.14	38.07 ± 15.25	0.540

*Statistically significant at p<0.05. Values are mean ± standard deviation or median (interquartile range). Nine distances are reported in millimeters; AP-FRAP, Sliding Angle and CL-OD are in degrees. TL-OD (R/L), right/left transverse-ligament attachment process to odontoid midline; C1 (A)-OD, posterior C1 anterior-arch border to anterior odontoid border; C1 (P)-OD, posterior odontoid border to anterior C1 posterior-arch border; C1 (P)-OFP, posteroinferior fracture endpoint to anterior midpoint of C1 posterior arch; C2 (P)-OFP, posteroinferior fracture endpoint to midpoint of C2 lamina anterior border; C1 (P)-OIP, posterior C2 inferior-endplate tip to anterior midpoint of C1 posterior arch; C1 (A)-C2P, anterior C2-lamina midpoint to posterior midpoint of C1 anterior arch; OD FLIP, anteroposterior odontoid translation; AP-FRAP, angle defined by the clival apical-ligament attachment, anterior fracture endpoint and posterior C2 body border; Sliding Angle, angle between anterior odontoid-fragment and anterior C2 body lines; CL-OD, clivus–odontoid angle. Anatomical nomenclature is based on Ogden et al (reference 12 in the manuscript).

On injury CT, OD FLIP was 3.10 mm (IQR 2.35–4.78; range 0.1–12.6; n=32) in the operative group and 2.00 mm (IQR 1.50–2.80; range 0–6.2; n=53) in the non-operative group (Table 2). These CT values describe a separate baseline measurement and should not be equated with signed lateral-radiographic fracture displacement.

### Serial Radiographic Evolution

Injury lateral radiographs were available for 20 of 33 operative and 42 of 53 non-operative patients. Signed fracture displacement was −3.0 mm (IQR −5.0 to −1.75) and −1.0 mm (IQR −3.0 to 0), respectively. The corresponding absolute displacement was 4.0 mm (IQR 3.0–5.0; range 1–9) and 2.0 mm (IQR 1.0–3.0; range 0–7). Thus, the signed group medians did not describe absolute displacement magnitude or imply minimal displacement in every patient. Different patients contributed measurements at different timepoints; changes in group medians should not be interpreted as paired individual changes.

At group level, no statistically significant longitudinal change in fracture alignment was detected between the first postoperative assessment and 6 months. Median fracture displacement remained −1 mm at all timepoints, with no significant overall change (p=0.920). Median fracture angulation likewise remained −5° at all timepoints, with no significant overall change (p=0.094) or difference between 1 week and 6 months after Holm adjustment (adjusted p=0.091; Table 3 and Figure 3).

**Table 3.** Serial Post-treatment Fracture Alignment

Parameter	Group	1 week, median (IQR)	6 weeks, median (IQR)	6 months, median (IQR)	Overall time effect, p	1 week vs 6 months, adjusted p
Fracture displacement, mm	Operative	−1.0 (−2.0 to 1.0)	−1.0 (−2.0 to 0.0)	−1.0 (−2.0 to 1.0)	0.920	1.000
Fracture displacement, mm	Non-operative	−1.25 (−3.0 to −0.6)	−2.0 (−3.0 to −0.5)	−2.0 (−3.0 to −0.5)	0.679	1.000
Fracture angulation, °	Operative	−5.0 (−11.3 to 5.3)	−5.0 (−14.5 to 0.8)	−5.0 (−14.0 to 0.5)	0.094	0.091
Fracture angulation, °	Non-operative	−10.5 (−19.8 to −4.0)	−15.0 (−22.0 to −3.5)	−13.0 (−24.0 to −1.0)	0.714	1.000

The 1-week, 6-week and 6-month values describe post-treatment alignment, not the original injury displacement. Operative follow-up measurements therefore represent residual alignment after reduction and fixation. Values are signed measurements and are presented as median (IQR). Overall p values were obtained from linear mixed-effects models with timepoint as a fixed effect and patient ID as a random intercept. The final column reports the Holm-adjusted comparison between 1 week and 6 months. IQR, interquartile range.

**Figure 3.**
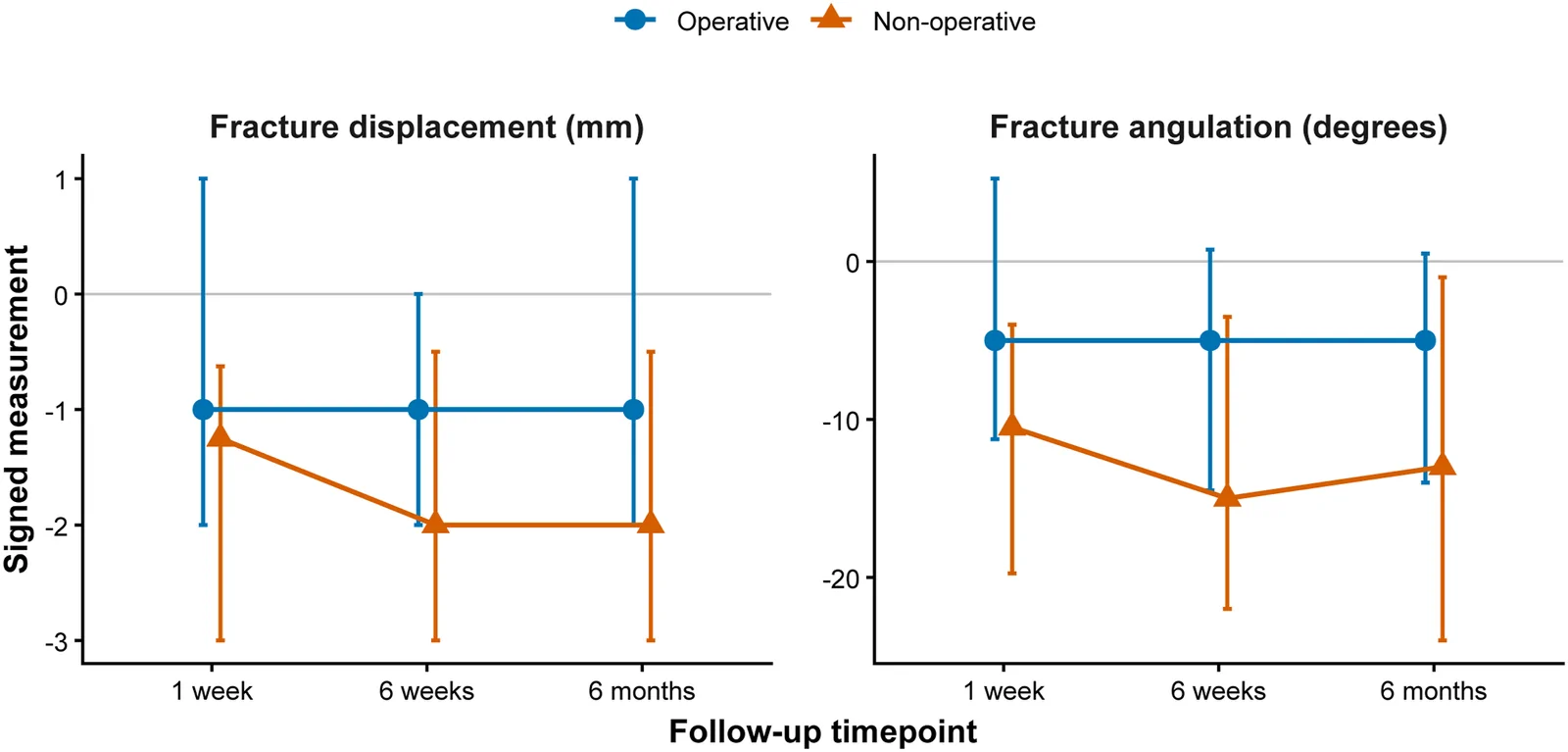
Serial fracture displacement and angulation after definitive treatment. Signed displacement (mm) and angulation (degrees) are shown at 1 week, 6 weeks, and 6 months after treatment initiation for the operative and non-operative groups.

Among 32 operative patients with postoperative measurements, no significant timepoint-by-technique interaction was detected for displacement (p=0.092) or angulation (p=0.823). No between-technique difference was significant at any individual timepoint after Holm adjustment. Analyses using absolute values produced similar results (Supplementary Table S7).

Among non-operatively treated patients with available follow-up imaging, neither fracture displacement (overall p=0.679) nor fracture angulation (overall p=0.714) changed significantly through 6 months.

No consistent deterioration in broader cervical alignment was observed in either treatment group. Odontoid incidence was the only parameter that changed significantly in the non-operative group (adjusted p=0.023), and fracture angulation at 6 weeks was the only significant between-group difference after adjustment for multiple testing. Detailed results are provided in Table 3 and Supplementary Tables S3–S5.

### Mortality and Survival Analysis

By the administrative censoring date of 1 February 2026, 50 deaths had occurred. Kaplan–Meier analysis showed no significant difference in survival between operative and non-operative management (log-rank p=0.198; Figure 4A).

**Figure 4.**
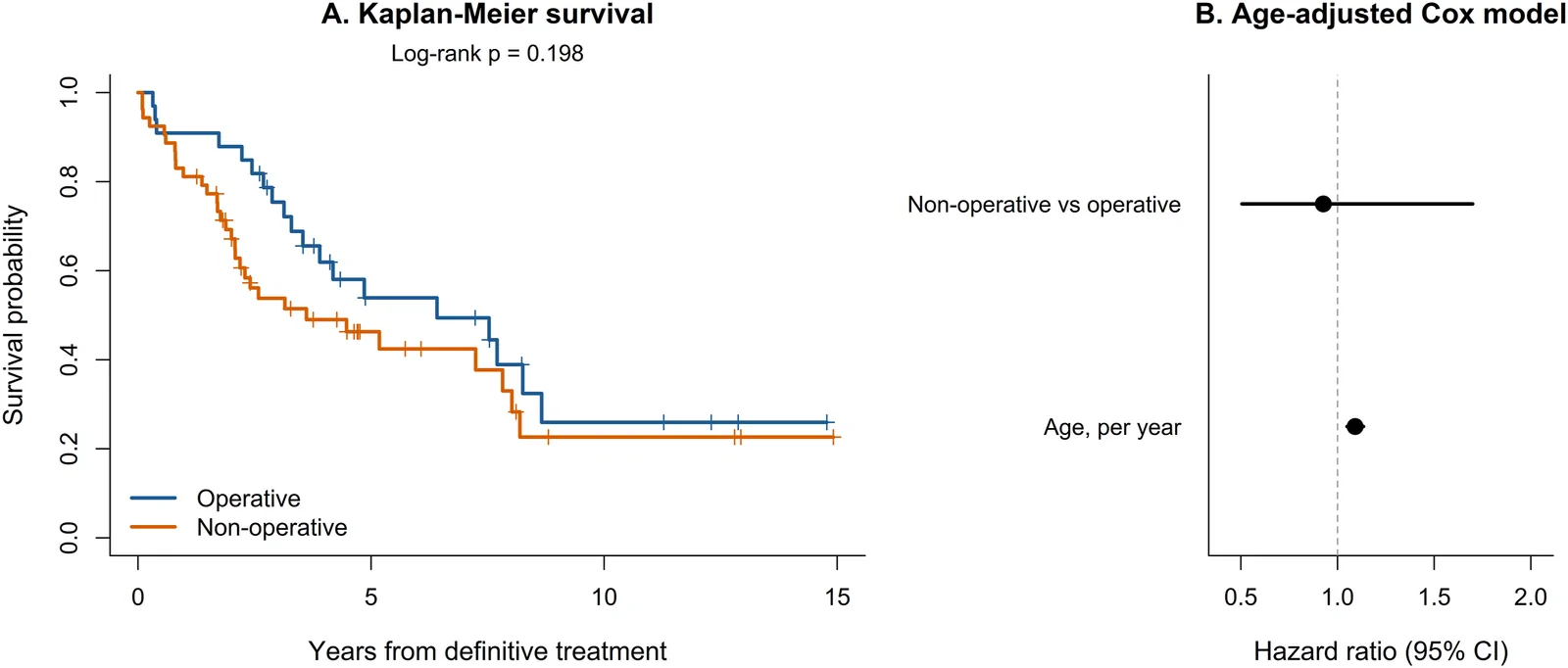
All-cause survival according to definitive treatment. (A) Kaplan–Meier survival curves for operative and non-operative management. Tick marks indicate censored observations; the log-rank p value was 0.198. (B) Age-adjusted Cox regression model showing hazard ratios for non-operative versus operative management and for each additional year of age at definitive treatment initiation. Points represent hazard ratios and horizontal lines represent 95% confidence intervals; the dashed vertical line denotes a hazard ratio of 1.0. Time was measured from definitive treatment initiation to death or administrative censoring on 1 February 2026. CI, confidence interval; HR, hazard ratio

In the age-adjusted Cox model, non-operative management was not significantly associated with mortality compared with operative treatment (HR 0.93, 95% CI 0.51–1.70; p=0.806). Increasing age at definitive treatment initiation was independently associated with mortality (HR 1.09 per year, 95% CI 1.05–1.13; p<0.0001; Figure 4B). Testing based on Schoenfeld residuals suggested potential non-proportionality for treatment group (p=0.041), although the global test was not significant (p=0.112). In a strict complete-case sensitivity analysis excluding the 10 patients for whom the baseline CT date was used as a proxy for treatment initiation, 76 patients (29 operative and 47 non-operative) and 45 deaths were analyzed. Results were similar: the treatment estimate remained non-significant (non-operative vs operative: HR 0.81, 95% CI 0.42–1.54; p=0.515), whereas increasing age remained associated with mortality (HR 1.11 per year, 95% CI 1.06–1.15; p<0.0001; Supplementary Table S2).

At 6 months, cumulative mortality was 3/33 (9.1%; 95% CI 3.0–25.6) in the operative group and 4/53 (7.5%; 95% CI 2.9–18.9) in the non-operative group. At 12 months, the corresponding values were 3/33 (9.1%; 95% CI 3.0–25.6) and 10/53 (18.9%; 95% CI 10.6–32.2; Table 4). No patient was censored before 12 months, so the observed proportions coincided with the Kaplan–Meier estimates. The six additional deaths between 6 and 12 months occurred in the non-operative group.

**Table 4.** Cumulative All-Cause Mortality During the First Year

Time from definitive treatment	Definitive treatment group	Cumulative deaths/initial cohort	Mortality, % (95% CI)	Patients at risk
6 months	Operative	3/33	9.1 (3.0–25.6)	30
6 months	Non-operative	4/53	7.5 (2.9–18.9)	49
12 months	Operative	3/33	9.1 (3.0–25.6)	30
12 months	Non-operative	10/53	18.9 (10.6–32.2)	43

Time was measured from the same operational definitive-treatment initiation date used in the primary survival analysis: the recorded 1-week radiograph date minus 7 days, with baseline CT-date fallback when unavailable. Six and 12 months correspond to 0.5 and 1 year (365.25 days per year). Mortality is 1 minus Kaplan–Meier survival; pointwise 95% confidence limits use the log–log transformation. No patient was censored before 12 months, so the estimates equal the observed death proportions. Death counts are cumulative from treatment initiation. Patients at risk were alive and uncensored at the specified time. These are unadjusted descriptive estimates. CI, confidence interval; CT, computed tomography.

During the first 12 months, 10 deaths occurred over 577 person-months in the non-operative group and 3 deaths over 373 person-months in the operative group. During the first 24 months, the corresponding values were 16 deaths over 1,051 person-months and 4 deaths over 730 person-months, respectively. In exploratory screening analyses, no baseline CT-derived morphologic parameter was significantly associated with mortality after false-discovery-rate correction at any examined time horizon. Detailed results are provided in Supplementary Table S6.

The proportion of operatively treated patients decreased from 56.2% in 2010–2014 to 44.4% in 2015–2019 and 27.9% in 2020–2025 (Supplementary Table S1). Adding calendar year of definitive treatment to the age-adjusted Cox model did not materially alter the treatment estimate (HR 0.88, 95% CI 0.48–1.63; p=0.688). Calendar year was not independently associated with mortality (HR 1.06 per year, 95% CI 0.96–1.16; p=0.235). Full model estimates are provided in Supplementary Table S2.

## Discussion

### Principal Findings

The present study yielded two principal observations. First, among patients with available follow-up imaging, operative reduction was maintained, and no statistically significant progressive displacement or angulation was detected after non-operative management through 6 months. Second, increasing age was independently associated with all-cause mortality, whereas definitive treatment strategy was not.

### Serial Radiographic Evolution

Serial follow-up showed no detectable loss of operative reduction between the first postoperative assessment and 6 months. Likewise, among non-operatively treated patients with available imaging, no statistically significant progressive fracture displacement or angulation was detected. Broader cervical alignment also showed no consistent deterioration in either group. These findings address the clinically relevant post-treatment course rather than merely reflecting the immediate correction achieved through surgery.

Injury displacement was often modest in this selected cohort, although the ranges included larger displacements. Treatment allocation incorporated the clinical presentation and physiological fitness rather than a prespecified displacement threshold. The post-treatment findings therefore describe the selected patients with available imaging and should not be generalized to markedly displaced fractures or used to establish indications for either treatment strategy.

The observed stability is consistent with previous reports that selected older adults may maintain fracture alignment despite incomplete osseous union and that stable fibrous nonunion may represent an acceptable radiographic endpoint.^13-18^ Surgery nevertheless provides higher fusion rates than non-operative treatment.^2,19^ Because the present study evaluated static alignment rather than osseous union or dynamic stability, the findings should not be interpreted as evidence of equivalence between treatment strategies. Incomplete radiographic follow-up, particularly at 6 months in the non-operative group, further limits this interpretation. Moreover, without pain, functional, or patient-reported outcomes, radiographic stability cannot be assumed to represent clinical success.^20,21^

Within the operative cohort, exploratory analyses did not identify a statistically significant difference in the longitudinal course of displacement or angulation between anterior odontoid screw fixation and posterior C1–2 fixation. However, the small technique-specific subgroups, incomplete serial imaging, and limited precision preclude excluding clinically relevant differences or interpreting the absence of statistical significance as evidence of equivalence.

### Mortality, Treatment Selection, and Secular Trends

After adjustment for age, definitive treatment strategy was not significantly associated with all-cause mortality, whereas increasing age remained a strong predictor. This is consistent with adjusted cohort studies and prospective comparative data that have not demonstrated a clear survival advantage for either strategy.^20,22,23^ In contrast, meta-analyses have reported lower pooled mortality after surgery,^2,19,24^ highlighting the difficulty of separating treatment effects from the preferential selection of younger and fitter patients for surgery.

Confounding by indication remains central. Although adjustment for age addressed the principal measured imbalance, treatment selection likely also reflected frailty, mobility, cognitive status, anesthetic risk, fracture stability, and patient goals. Operative treatment became less frequent over time. However, adding calendar year of definitive treatment did not materially change the treatment estimate, suggesting that secular changes alone did not explain the primary result. Potential non-proportionality indicates that the Cox estimate should be interpreted as an overall association across follow-up rather than a constant hazard ratio.

Because physiological fitness was one consideration in treatment selection, preferential selection of younger or fitter patients for surgery could favor the operative group in observed mortality comparisons even without a beneficial treatment effect. Age adjustment and the absence of statistically significant CCI or ECI differences do not establish comparable frailty or physiological reserve. Unmeasured fitness, functional status and anesthetic risk may influence mortality throughout follow-up, including the first 6 and 12 months.

The early descriptive results showed similar observed mortality at 6 months and a greater separation by 12 months. The small number of deaths and wide confidence intervals preclude a reliable causal interpretation of this pattern. These fixed-time summaries and the overall age-adjusted Cox estimate address different aspects of follow-up; neither establishes mortality equivalence or excludes a clinically relevant treatment effect.

Baseline CT morphology was broadly comparable between groups except for greater anterior–posterior odontoid translation in the operative group, and no CT-derived parameter was associated with mortality after correction for multiple testing. Fracture morphology may therefore be more relevant to mechanical instability or nonunion than to survival.^25^ Crossovers from initial non-operative management to surgery further illustrate that treatment may evolve in response to pain or radiographic changes, as also reported in previous series.^2,17^

### Clinical Implications

In neurologically intact older adults with isolated type II odontoid fractures, the clinically relevant observation was the post-treatment course: operative reduction was maintained through 6 months, while selected non-operatively treated patients with available imaging showed no statistically significant progressive displacement or angulation. These findings provide a real-world benchmark for patient counselling and radiographic surveillance, indicating that progressive deformity was not inevitable during the observed follow-up. These observational results cannot establish a survival advantage for either strategy and should be interpreted alongside mechanical and patient-centered considerations. The study does not establish equivalence or define indications for either strategy.

### Limitations

This retrospective single-center study is subject to selection bias and confounding by indication. Operatively treated patients were younger, and treatment selection likely reflected unmeasured differences in frailty, mobility, cognitive status, functional reserve, and patient goals that were not captured by the CCI or ECI. Residual confounding therefore remains despite age adjustment. The as-treated analysis describes outcomes after definitive treatment pathways rather than the causal effect of initial treatment assignment. Cause-specific mortality was unavailable, and the 15-year study period may have introduced secular changes despite adjustment for calendar year.

Classification by definitive treatment may also select patients who survived until surgery, particularly those who crossed over after initial non-operative management. Follow-up beginning at a reconstructed definitive-treatment date does not fully capture risk before that pathway and may introduce survivor selection. The early mortality estimates share these limitations and describe outcomes from definitive treatment rather than from injury or admission.

Radiographic follow-up was incomplete, particularly at 6 months after non-operative treatment, and the study assessed static alignment rather than osseous union or dynamic stability. The modest cohort and small technique-specific subgroups limit precision and preclude conclusions of equivalence. Pain, function, complications, follow-up neurological outcomes, patient-reported outcomes, and quality of life were unavailable, limiting the clinical interpretation of radiographic stability.

## Conclusion

In this 15-year single-center cohort of older adults with isolated type II odontoid fractures, operative reduction was maintained, and no statistically significant progressive displacement or angulation was detected after non-operative management through 6 months. Increasing age was independently associated with all-cause mortality, whereas no statistically significant association with definitive treatment strategy was detected after age adjustment. The study does not establish treatment superiority or equivalence and does not define indications for either strategy. Larger studies incorporating dynamic stability and patient-centered outcomes are warranted.

## Supplemental Material

Supplemental Material - Serial Radiographic Evolution and All-Cause Mortality in Older Adults with Isolated Type II Odontoid Fractures: A 15-Year Single-Center Cohort Study Supplemental material for Serial Radiographic Evolution and All-Cause Mortality in Older Adults with Isolated Type II Odontoid Fractures: A 15-Year Single-Center Cohort Study by Jan Gewiess, Domas Bubulis, Samuel Friedrich Schaible, Moritz Caspar Deml, Thomas Lustenberger and Christian Tinner in Global Spine Journal.

## Data Availability

The data that support the findings of this study are available from the corresponding author upon reasonable request, subject to institutional, ethical, and legal restrictions.*
