# Supplementary material for: Serial Radiographic Evolution and All-Cause Mortality in Older Adults with Isolated Type II Odontoid Fractures: A 15-Year Single-Center Cohort Study
Source: Global Spine J. 2026 Nov 6:21925682261507648. Online ahead of print. doi: 10.1177/21925682261507648 (PMC13743548; doi:10.1177/21925682261507648)
Supplement: Supplementary file 1 [file sj-pdf-1-gsj-10.1177_21925682261507648.pdf]

# Supplementary Material

**Supplementary Table S1**: Cohort characteristics by calendar period of definitive treatment

| Calendar period | Patients, n | Operative, n (%) | Non-operative, n (%) | Age at treatment initiation, years, median (IQR) | Deaths, n (%) |
|---|---|---|---|---|---|
| 2010–2014 | 16 | 9 (56.2%) | 7 (43.8%) | 75.0 (71.6–86.6) | 9 (56.2%) |
| 2015–2019 | 27 | 12 (44.4%) | 15 (55.6%) | 85.1 (77.2–88.9) | 22 (81.5%) |
| 2020–2025 | 43 | 12 (27.9%) | 31 (72.1%) | 82.3 (74.1–86.2) | 19 (44.2%) |
| Total | 86 | 33 (38.4%) | 53 (61.6%) | 82.3 (74.3–87.3) | 50 (58.1%) |

Values are n (%) or median (IQR). Treatment groups reflect definitive management. Deaths are all-cause mortality events recorded before administrative censoring on 1 February 2026. IQR, interquartile range.

**Supplementary Table S2**: Cox regression models for all-cause mortality

| Model | Variable | HR | 95% CI | p value |
|---|---|---|---|---|
| **Age-adjusted** | Non-operative vs operative | 0.93 | 0.51–1.70 | 0.806 |
| Age-adjusted | Age at treatment initiation, per year | 1.09 | 1.05–1.13 | <0.0001 |
| Complete-case age-adjusted | Non-operative vs operative | 0.81 | 0.42–1.54 | 0.515 |
| Complete-case age-adjusted | Age at treatment initiation, per year | 1.11 | 1.06–1.15 | <0.0001 |
| **Age + calendar year** | Non-operative vs operative | 0.88 | 0.48–1.63 | 0.688 |
| Age + calendar year | Age at treatment initiation, per year | 1.09 | 1.05–1.13 | <0.0001 |
| Age + calendar year | Calendar year of treatment, per year | 1.06 | 0.96–1.16 | 0.235 |

Operative management is the reference category. HRs for age and calendar year are expressed per one-year increase. The complete-case sensitivity model excluded the 10 patients for whom the baseline CT date was used as a proxy for treatment initiation and included 76 patients (29 operative and 47 non-operative) and 45 deaths. Proportional-hazards testing for the primary age-adjusted model yielded p=0.041 for treatment, p=0.202 for age, and p=0.112 globally. HR, hazard ratio; CI, confidence interval.

**Supplementary Table S3**: Radiographic measurement availability by definitive treatment group and timepoint

| Timepoint | Operative available / total | Non-operative available / total | Total available / total |
|---|---|---|---|
| Injury | 20/33 | 42/53 | 62/86 |
| 1 week | 28/33 | 46/53 | 74/86 |
| 6 weeks | 31/33 | 46/53 | 77/86 |
| 6 months | 27/33 | 25/53 | 52/86 |

Availability denotes the presence of follow-up radiographs permitting serial measurement at the specified timepoint. Denominators are 33 operative patients, 53 non-operatively treated patients, and 86 patients overall.

**Supplementary Table S4**: Longitudinal mixed-effects model results for radiographic parameters

| Radiographic parameter | Group | LMM overall time effect, p value | Main Holm-adjusted post hoc findings |
|---|---|---|---|
| **Odontoid fracture displacement** | Operative | 0.920 | No Holm-adjusted pairwise contrast was significant; 1 week versus 6 months, adjusted p=1.000. |
| Odontoid fracture displacement | Non-operative | 0.679 | No Holm-adjusted pairwise contrast was significant; 1 week versus 6 months, adjusted p=1.000. |
| **Odontoid fracture angulation** | Operative | 0.094 | No Holm-adjusted pairwise contrast was significant; 1 week versus 6 months, adjusted p=0.091. |
| Odontoid fracture angulation | Non-operative | 0.714 | No Holm-adjusted pairwise contrast was significant; 1 week versus 6 months, adjusted p=1.000. |
| **Odontoid incidence** | Operative | <0.001 | Odontoid incidence decreased from injury radiography to 1 week, 6 weeks, and 6 months; all Holm-adjusted p≤0.0011. |
| Odontoid incidence | Non-operative | 0.030 | Odontoid incidence increased from 1 week to 6 months; Holm-adjusted p=0.023. |
| **Odontoid tilt** | Operative | 0.984 | No Holm-adjusted pairwise contrast was significant. |
| Odontoid tilt | Non-operative | 0.245 | No Holm-adjusted pairwise contrast was significant. |
| **C2 slope** | Operative | 0.812 | No Holm-adjusted pairwise contrast was significant. |
| C2 slope | Non-operative | 0.727 | No Holm-adjusted pairwise contrast was significant. |
| **Gravity line-to-C2 distance** | Operative | 0.010 | Gravity line-to-C2 distance decreased from injury radiography to 1 week, 6 weeks, and 6 months; Holm-adjusted p=0.015, p=0.031, and p=0.031, respectively. |
| Gravity line-to-C2 distance | Non-operative | 0.326 | No Holm-adjusted pairwise contrast was significant. |
| **cSVA** | Operative | <0.001 | cSVA decreased from injury radiography to 6 weeks and 6 months; Holm-adjusted p=0.002 and p=0.002, respectively. |
| cSVA | Non-operative | 0.068 | No Holm-adjusted pairwise contrast was significant. |
| **C0–2 angle** | Operative | 0.018 | C0–2 angle decreased from injury radiography to 1 week; Holm-adjusted p=0.018. |
| C0–2 angle | Non-operative | 0.590 | No Holm-adjusted pairwise contrast was significant. |
| **C2–7 angle** | Operative | 0.564 | No Holm-adjusted pairwise contrast was significant. |
| C2–7 angle | Non-operative | 0.827 | No Holm-adjusted pairwise contrast was significant. |
| **T1 slope** | Operative | 0.464 | No Holm-adjusted pairwise contrast was significant. |
| T1 slope | Non-operative | 0.723 | No Holm-adjusted pairwise contrast was significant. |

Mixed-effects models of fracture displacement and angulation included the post-treatment assessments at 1 week, 6 weeks, and 6 months. Exploratory models of the remaining radiographic parameters included available injury and follow-up measurements. All models included timepoint as a fixed effect and patient ID as a random intercept. Holm adjustment was applied to targeted post hoc comparisons. Complete-case analyses included 22 operative and 21 non-operative patients and were concordant for displacement (Friedman p=0.865 in the operative group and p=0.687 in the non-operative group) and angulation (p=0.798 and p=0.675, respectively); no Holm-adjusted paired comparison was significant. LMM, linear mixed-effects model; cSVA, cervical sagittal vertical axis.

**Supplementary Table S5**: Between-group Radiographic Comparisons at Injury and Follow-up Timepoints

| Parameter | Timepoint | Operative, median (IQR) | Non-operative, median (IQR) | p value | Holm-adjusted p |
|---|---|---|---|---|---|
| **Odontoid fracture displacement** | Injury | −3.00 (−5.00 to −1.75), n=20 | −1.00 (−3.00 to 0.00), n=42 | 0.031 | 0.123 |
| Odontoid fracture displacement | 1 week | −1.00 (−2.00 to 1.00), n=28 | −1.25 (−3.00 to −0.62), n=46 | 0.109 | 0.218 |
| Odontoid fracture displacement | 6 weeks | −1.00 (−2.00 to 0.00), n=31 | −2.00 (−3.00 to −0.50), n=46 | 0.071 | 0.214 |
| Odontoid fracture displacement | 6 months | −1.00 (−2.00 to 1.00), n=27 | −2.00 (−3.00 to −0.50), n=25 | 0.178 | 0.218 |
| **Odontoid fracture angulation** | Injury | −7.00 (−16.25 to 1.00), n=20 | −9.00 (−22.00 to −3.25), n=42 | 0.468 | 0.468 |
| Odontoid fracture angulation | 1 week | −5.00 (−11.25 to 5.25), n=28 | −10.50 (−19.75 to −4.00), n=46 | 0.017 | 0.052 |
| Odontoid fracture angulation | 6 weeks | −5.00 (−14.50 to 0.75), n=31 | −15.00 (−22.00 to −3.50), n=46 | 0.008 | 0.030 |
| Odontoid fracture angulation | 6 months | −5.00 (−14.00 to 0.50), n=27 | −13.00 (−24.00 to −1.00), n=25 | 0.088 | 0.176 |
| **Odontoid incidence** | Injury | 27.00 (19.75 to 34.00), n=20 | 23.50 (19.00 to 28.85), n=42 | 0.256 | 0.354 |
| Odontoid incidence | 1 week | 20.00 (14.25 to 26.00), n=28 | 23.00 (18.00 to 28.75), n=46 | 0.177 | 0.354 |
| Odontoid incidence | 6 weeks | 22.00 (15.00 to 26.00), n=31 | 26.00 (19.25 to 29.00), n=46 | 0.033 | 0.099 |
| Odontoid incidence | 6 months | 20.00 (15.00 to 24.00), n=27 | 28.00 (23.00 to 31.00), n=25 | 0.017 | 0.068 |
| **Odontoid tilt** | Injury | 7.50 (3.75 to 17.75), n=20 | 7.00 (4.00 to 13.00), n=42 | 0.821 | 1.000 |
| Odontoid tilt | 1 week | 7.50 (3.00 to 17.00), n=28 | 8.50 (4.25 to 14.75), n=46 | 0.776 | 1.000 |
| Odontoid tilt | 6 weeks | 6.00 (3.00 to 14.50), n=31 | 10.00 (3.00 to 14.00), n=46 | 0.670 | 1.000 |
| Odontoid tilt | 6 months | 7.00 (3.00 to 15.50), n=27 | 5.00 (3.00 to 8.00), n=25 | 0.398 | 1.000 |
| **C2 slope** | Injury | 22.50 (17.75 to 27.25), n=20 | 22.00 (14.25 to 26.23), n=42 | 0.519 | 1.000 |
| C2 slope | 1 week | 23.00 (17.00 to 28.00), n=28 | 24.00 (14.75 to 30.00), n=46 | 0.915 | 1.000 |
| C2 slope | 6 weeks | 23.00 (16.50 to 29.00), n=31 | 24.00 (15.25 to 29.50), n=46 | 0.600 | 1.000 |
| C2 slope | 6 months | 23.00 (15.50 to 28.50), n=27 | 23.00 (18.00 to 30.00), n=25 | 0.871 | 1.000 |
| **Gravity line-to-C2 distance** | Injury | 66.00 (56.50 to 70.25), n=20 | 60.00 (55.00 to 66.00), n=41 | 0.331 | 0.993 |
| Gravity line-to-C2 distance | 1 week | 57.00 (52.50 to 64.00), n=28 | 58.50 (52.25 to 65.75), n=46 | 0.489 | 0.993 |
| Gravity line-to-C2 distance | 6 weeks | 57.00 (54.00 to 65.50), n=31 | 58.00 (52.50 to 66.75), n=46 | 0.775 | 0.993 |
| Gravity line-to-C2 distance | 6 months | 60.00 (54.00 to 64.50), n=27 | 63.00 (56.00 to 68.00), n=25 | 0.179 | 0.718 |
| **cSVA** | Injury | 85.00 (75.50 to 91.00), n=19 | 78.00 (73.00 to 88.00), n=41 | 0.332 | 0.947 |
| cSVA | 1 week | 82.00 (72.75 to 86.75), n=28 | 76.00 (68.25 to 84.00), n=46 | 0.169 | 0.677 |
| cSVA | 6 weeks | 80.00 (73.50 to 85.50), n=31 | 78.50 (70.25 to 82.00), n=46 | 0.316 | 0.947 |
| cSVA | 6 months | 80.00 (71.50 to 85.50), n=27 | 79.00 (74.00 to 84.00), n=25 | 0.748 | 0.947 |
| **C0–2 angle** | Injury | 51.00 (46.00 to 57.00), n=20 | 50.00 (45.00 to 54.00), n=42 | 0.497 | 1.000 |
| C0–2 angle | 1 week | 48.50 (40.75 to 53.75), n=28 | 53.00 (45.25 to 59.75), n=46 | 0.160 | 0.638 |
| C0–2 angle | 6 weeks | 48.00 (43.50 to 55.50), n=31 | 51.50 (41.25 to 59.50), n=46 | 0.508 | 1.000 |
| C0–2 angle | 6 months | 48.00 (40.00 to 55.50), n=27 | 52.00 (44.00 to 59.00), n=25 | 0.507 | 1.000 |
| **C2–7 angle** | Injury | 13.00 (9.00 to 24.00), n=19 | 17.00 (11.75 to 27.25), n=40 | 0.505 | 1.000 |
| C2–7 angle | 1 week | 11.00 (5.00 to 21.25), n=28 | 16.25 (10.00 to 24.00), n=46 | 0.202 | 0.806 |
| C2–7 angle | 6 weeks | 17.00 (12.00 to 24.50), n=31 | 17.00 (9.00 to 25.00), n=45 | 0.583 | 1.000 |
| C2–7 angle | 6 months | 17.00 (10.00 to 25.00), n=27 | 15.00 (10.00 to 21.00), n=25 | 0.728 | 1.000 |
| **T1 slope** | Injury | 40.00 (32.75 to 44.75), n=18 | 35.00 (28.00 to 45.75), n=38 | 0.351 | 1.000 |
| T1 slope | 1 week | 34.50 (30.00 to 42.00), n=28 | 39.00 (32.25 to 45.00), n=46 | 0.469 | 1.000 |
| T1 slope | 6 weeks | 35.00 (29.50 to 46.00), n=31 | 36.00 (31.00 to 45.00), n=45 | 0.723 | 1.000 |
| T1 slope | 6 months | 38.50 (28.75 to 49.25), n=26 | 37.00 (31.00 to 43.00), n=25 | 0.660 | 1.000 |

Displacement, gravity line-to-C2 distance, and cSVA are reported in millimeters; angular parameters are reported in degrees. Values are signed measurements and are presented as median (IQR), with the available n shown in each cell. Unadjusted p values compare treatment groups at each timepoint; Holm-adjusted p values are corrected across the four timepoints within each parameter. IQR, interquartile range; cSVA, cervical sagittal vertical axis. For available injury radiographs, absolute displacement was 4.0 mm (IQR 3.0–5.0; range 1–9; n=20) in the operative group and 2.0 mm (IQR 1.0–3.0; range 0–7; n=42) in the non-operative group. Signed values retain measurement direction; the absolute summaries describe magnitude. Follow-up timepoints were measured after definitive treatment initiation.

**Supplementary Table S6**: Exploratory screening of baseline CT morphologic parameters for association with mortality at predefined time horizons

| Time horizon | Subgroup | Parameter | n | Point-biserial r | p value | BH-adjusted p | Spearman rho | Spearman p |
|---|---|---|---|---|---|---|---|---|
| 3 months | Overall | TL-OD (L) | 85 | 0.198 | 0.069 | 0.413 | 0.152 | 0.165 |
| 3 months | Overall | C1 (P)-OD | 85 | 0.162 | 0.138 | 0.554 | 0.175 | 0.108 |
| 3 months | Overall | CL-OD | 84 | -0.208 | 0.058 | 0.413 | -0.232 | 0.034 |
| 3 months | Non-operative | TL-OD (L) | 53 | 0.219 | 0.115 | 0.386 | 0.163 | 0.244 |
| 3 months | Non-operative | C1 (P)-OD | 53 | 0.214 | 0.124 | 0.386 | 0.224 | 0.106 |
| 3 months | Non-operative | OD FLIP | 53 | 0.211 | 0.129 | 0.386 | 0.222 | 0.111 |
| 3 months | Non-operative | CL-OD | 53 | -0.228 | 0.101 | 0.386 | -0.272 | 0.049 |
| 6 months | Overall | C1 (P)-OD | 85 | 0.185 | 0.091 | 0.545 | 0.144 | 0.188 |
| 6 months | Overall | CL-OD | 84 | -0.197 | 0.073 | 0.545 | -0.234 | 0.032 |
| 6 months | Non-operative | C1 (P)-OD | 53 | 0.202 | 0.147 | 0.720 | 0.210 | 0.131 |
| 6 months | Non-operative | CL-OD | 53 | -0.236 | 0.088 | 0.720 | -0.294 | 0.032 |
| 1 year | Overall | TL-OD (R) | 85 | 0.199 | 0.068 | 0.306 | 0.187 | 0.086 |
| 1 year | Overall | C1 (P)-OD | 85 | 0.219 | 0.044 | 0.306 | 0.217 | 0.046 |
| 1 year | Overall | C1 (P)-OFP | 85 | 0.166 | 0.129 | 0.388 | 0.099 | 0.370 |
| 1 year | Overall | CL-OD | 84 | -0.194 | 0.077 | 0.306 | -0.236 | 0.030 |
| 1 year | Non-operative | TL-OD (R) | 53 | 0.262 | 0.058 | 0.352 | 0.257 | 0.063 |
| 1 year | Non-operative | C1 (P)-OD | 53 | 0.261 | 0.059 | 0.352 | 0.293 | 0.033 |
| 1 year | Non-operative | CL-OD | 53 | -0.212 | 0.127 | 0.443 | -0.273 | 0.048 |
| 2 years | Overall | C1 (P)-OD | 85 | 0.236 | 0.030 | 0.177 | 0.225 | 0.039 |
| 2 years | Overall | CL-OD | 84 | -0.239 | 0.029 | 0.177 | -0.229 | 0.036 |
| 2 years | Non-operative | C1 (P)-OD | 53 | 0.293 | 0.033 | 0.169 | 0.294 | 0.032 |
| 2 years | Non-operative | C1 (A)-C2P | 53 | 0.280 | 0.042 | 0.169 | 0.288 | 0.037 |
| 2 years | Non-operative | CL-OD | 53 | -0.303 | 0.027 | 0.169 | -0.321 | 0.019 |

Mortality at each horizon was defined as recorded all-cause death within that interval after definitive treatment initiation. Only associations with an unadjusted point-biserial $p<0.15$ are displayed. Benjamini–Hochberg correction was performed across all 12 CT parameters within each time horizon and subgroup; no association remained significant after correction. Correlations used available cases for each parameter, and Spearman correlations were examined as sensitivity analyses. BH, Benjamini–Hochberg; CT, computed tomography. TL-OD (R/L), right/left transverse-ligament attachment-to-odontoid distance; C1 (P)-OD, posterior odontoid-to-posterior atlas-arch interval; C1 (P)-OFP, posterior atlas arch-to-posteroinferior fracture-point distance; C1 (A)-C2P, anterior atlas arch-to-C2 lamina distance; OD FLIP, anteroposterior odontoid translation; CL-OD, clivus–odontoid angle. Distances are in millimeters and CL-OD is in degrees. The full CT measurement descriptions are also provided in Table 2.

**Supplementary Table S7**: Exploratory comparison of serial fracture alignment by operative technique

**Panel A. Technique-specific descriptive statistics and model-based contrasts**

| Outcome | Timepoint | Anterior, n; median (IQR) | Posterior, n; median (IQR) | Posterior − anterior estimate (95% CI) | Holm-adjusted p |
|---|---|---|---|---|---|
| **Displacement** | 1 week | 17; −1.0 (−2.0 to 1.0) | 11; −1.0 (−2.0 to 1.0) | 0.18 (−1.79 to 2.15) | 0.858 |
| Displacement | 6 weeks | 19; −1.0 (−2.0 to 2.0) | 12; −1.0 (−2.0 to −0.88) | −1.14 (−3.03 to 0.75) | 0.563 |
| Displacement | 6 months | 17; −1.5 (−3.0 to −0.5) | 10; −1.0 (−1.0 to 2.0) | 1.35 (−0.67 to 3.37) | 0.563 |
| **Angulation** | 1 week | 17; −7.0 (−13.0 to −4.0) | 11; −2.0 (−7.5 to 7.0) | 3.61 (−6.31 to 13.53) | 0.636 |
| Angulation | 6 weeks | 19; −9.0 (−16.0 to 2.0) | 12; −1.0 (−8.75 to 0.13) | 5.26 (−4.41 to 14.93) | 0.636 |
| Angulation | 6 months | 17; −6.0 (−18.0 to 0.0) | 10; −2.5 (−7.0 to 0.5) | 6.36 (−3.72 to 16.43) | 0.636 |

**Panel B. Interaction and sensitivity analyses**

| Outcome | Analysis | Comparison | Chi-square | df | p value | Observations | Patients |
|---|---|---|---|---|---|---|---|
| **Displacement** | Signed values | Timepoint × technique | 4.78 | 2 | 0.092 | 86 | 32 |
| Angulation | Signed values | Timepoint × technique | 0.39 | 2 | 0.823 | 86 | 32 |
| **Displacement** | Absolute-value sensitivity | Timepoint × technique | 1.16 | 2 | 0.560 | 86 | 32 |
| Angulation | Absolute-value sensitivity | Timepoint × technique | 0.78 | 2 | 0.677 | 86 | 32 |
| **Displacement** | Influence sensitivity; ID 78 excluded | Timepoint × technique | 5.27 | 2 | 0.072 | 83 | 31 |
| Angulation | Influence sensitivity; ID 33 excluded | Timepoint × technique | 0.58 | 2 | 0.750 | 83 | 31 |

Displacement is reported in millimeters and angulation in degrees. Panel A presents signed values as median (IQR) and model-based posterior-minus-anterior estimates with 95% CIs. Holm adjustment was applied across the three postoperative timepoints within each outcome. Panel B reports likelihood-ratio tests of the timepoint-by-technique interaction from mixed-effects models with patient-specific random intercepts. Absolute-value analyses assessed measurement magnitude irrespective of direction. CI, confidence interval; df, degrees of freedom; IQR, interquartile range.
